# Computer and Internet use among Undergraduate Medical Students in Iran

**DOI:** 10.12669/pjms.305.5355

**Published:** 2014

**Authors:** Ali Ayatollahi, Jamshid Ayatollahi, Fatemeh Ayatollahi, Reza Ayatollahi, Seyed Hossein Shahcheraghi

**Affiliations:** 1 Ali Ayatollahi, MSc Student of Optometry, Shahid Beheshti University of Medical Sciences and Health Services, Tehran, Iran.; 2 Jamshid Ayatollahi, Infectious and Tropical Diseases Research Center, Shahid Sadoughi University of Medical Sciences, Yazd, Iran.; 3 Fatemeh Ayatollahi, Endodontic, Shahid Sadoughi University of Medical Sciences, Yazd, Iran.; 4 Reza Ayatollahi, Dentistry Student, International Campus, Shahid Sadoughi University of Medical Sciences, Yazd, Iran.; 5 Seyed Hossein Shahcheraghi, Infectious and Tropical Diseases Research Center, Shahid Beheshti University of Medical Sciences and Health Services, Tehran, Iran.

**Keywords:** Computer, Internet, Medical students

## Abstract

***Objective: ***Although computer technologies are now widely used in medicine, little is known about its use among medical students in Iran. The aim of this study was to determine the competence and access to computer and internet among the medical students.

***Methods: ***In this descriptive study, medical students of Shahid Sadoughi University of Medical Science, Yazd, Iran from the fifth years were asked to answer a questionnaire during a time-tabled lecture slot. The chi-square test was used to compare the frequency of computer and internet use between the two genders, and the level of statistical significance for all test was set at 0.05.

***Results:*** All the students have a personal computer and internet access. There were no statistically significant differences between men and women for the computer and internet access, use wireless device to access internet, having laptop and e-mail address and the difficulties encountered using internet. The main reason for less utilization of internet was slow speed of data transfer.

***Conclusions: ***Because of the wide range of computer skills and internet information among medical students in our institution, a single computer and internet course for all students would not be useful nor would it be accepted.

## INTRODUCTION

Over the last several years studies have shown the use of computer and internet by medical student is increasing. The major goals of education are to encourage medical students to increase their knowledge of medical science and maintain update knowledge by becoming life-long learners. The internet is cost-effective and fast.^1^^-^^3^ The information can be accessed from any source with an internet connection and with no time limitation.^4^ Internet medical sites containing up-to-date clinical, laboratory, radiographic, treatment, prophylaxis and outcome data of diseases create an environment for medical students to study at their own pace.^5^Availability of medical full- text articles and other databases may have a major impact on the selection of information resources among students. Students can review cases reports and can have the opportunity to learn about different views on controversial topics.^6^ In addition, the computer and internet can motivate medical students to undertake research and help them to develop skills in collecting and analyzing data.^7^^,^^8^

The aim of this study was to find out how electronic information resources are utilized by medical students, and find out how frequently they access to the internet, find out the time using the internet, determine views on the accuracy and relevance of internet information and find out possible barriers to increased use of the internet.

## METHODS

This is a descriptive, cross sectional questionnaire survey. A two-page structured questionnaire was distributed to the fifth year clinical undergraduate students studying at Shahid Sadoughi University of Medical Science, Yazd, Iran. A questionnaire consisting of multiple choice questions was prepared regarding computer access, computer training and skills, computer activities, the methods students prefer to access medical literature, where they get access to internet, reasons for internet uses factors restricting students from using the internet, sources on the internet preferred by students to gain information on medical subjects, and how often they are able to find medical subjects in Farsi and English site. 

The content validity of the questionnaire was obtained through the review of other questionnaires and three experts reviewed the forms to establish face validity. Questionnaires were distributed to all students during a time-tabled lecture slot, and were collected after the students finished filling them out. The propose of this study was explained, and students were asked not to write their names in the questionnaire. The students were also reminded that they could choose more than one option in some of the questions. The data were processed and analyzed using SPSS version 15 for statistical package .The chi-square test was used to compare the answers from gender. The results were considered as statistically significant if the P value was less than 0.05.

## RESULTS

One hundred nine students (65 female and 44 male) were asked to fill the questionnaire. The entire questionnaire distributed were filled out and collected. The participants' age ranged from 22 to 26 years, with mean age ± SD: 23.34±1.21 years. 

Table-I summarizes the students' responses to the questionnaire in relation to the following categories: computer access, computer skills and training, laptop access, how familiar they were with computer, how old were they when they had computer and printer access.

**Fig.1:**
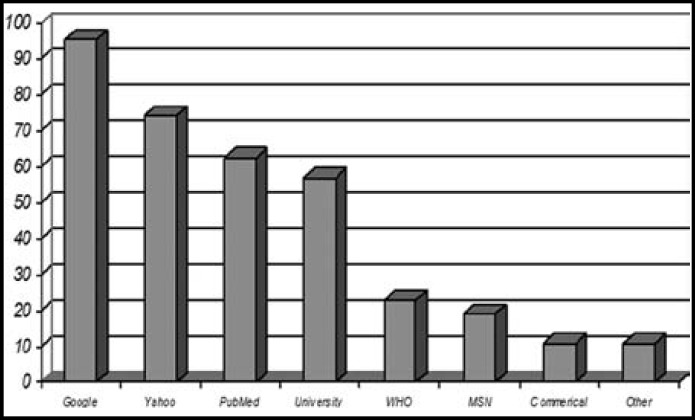
Internet sites used by medical students to access non medical subjects

As shown in Table II, 100% of medical students reported that they used the internet with various frequencies. While 61.5% of the female students used the internet at least once a week, this frequency was 38.5% for male students (PV=0.401). Fig.1 shows the internet sites that medical students preferred to use to access non medical information.

**Fig.2:**
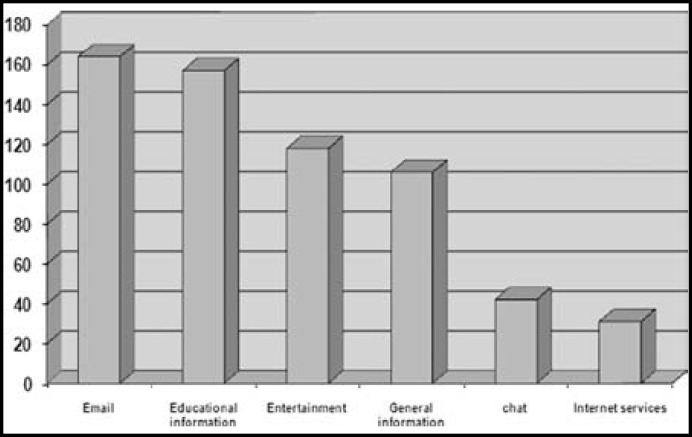
Frequencies of different kind of internet utilization

The internet sites that medical students used more to access subjects on medical topics was 60.6% Pub Med, 34.9% medical journals and 21.1% others. 78.9% of the medical students declare using internet for Text, 67 Clinical photographs, 17.4 Radiographic slide and 17.4% for film. 59.6% of the female students used the internet for general information, this frequency was 40.4% for male students (PV=0.023) (Fig.2). The frequency of medical subjects use in Farsi language and English-language sites is presented in Table III.

Replying to the question regarding reasons for less utilization of internet, slow speed of data transfer was mentioned by most (47.7%). Other reasons mentioned included lack of time (40.4) , do not know how to use the internet (18.3), cost (17.4%), difficulty in finding a computer with internet access (15.7%) and others (8.3%).

## DISCUSSION

Despite numerous worldwide studies performed about the use of computer and internet by medical students, few studies have been conducted in Iran. Following the study performed about the use of computer and internet by dental students in Shahid Sadoughi University of Medical Sciences, this study was conducted among medical students in Shahid Sadoughi University of Medical Sciences.^5^

According to our data, the rate of computer usage among medical students is competitive with dental students and medical students in other studies. For example, in our study all medical students (100%) had personal computer (P.C) which was more than Indian (79%), Australian (94%), Finish (83%), Jordanian (73.9%), English (72%) and Moroccan students (71.1%).^6^^,^^9^^-^^14^ Also, all medical students (100%) had access to computer in university which is the same as Jordanian students.53.3% of Shahid Sadoughi University of Medical Science students had personal laptop but 30% of Moroccan medical students had personal laptop and this difference was significant.

In this study 21.1% of students had access to university’s printer and 35.8% of them had this facility in their homes. Compared to Jordanian study, 100% of them had university printer access and 48.5% of them had home access to printer, which indicates Jordanian students had better facilities compared to Iranian students.^10^

**Table-I:** Questionnaire items on computer, laptop and printer access and computer skills and training

***Questions***	***%***	***No***
1-Do you have access to computer?		
Yes	100	109
No	0	0
2-If yes, please answer where?		
At university	91.7	100
At home	87.2	95
Internet cafe	9.2	10
Others	1.8	2
3-How would you describe the availability and access of the computer?		
Very good	35.8	38
Good	33	35
Adequate	26.4	28
Poor	4.7	5
Very poor	2.8	3
4-Do you have Laptop?		
Yes	53.2	58
No	46.8	51
5- Do you have access to printer?		
Yes	42.5	47
No	57.5	62
6-If yes, please answer the following:		
At home	35.8	39
At university	33.9	37
Internet cafe	17.4	23
Others	21.1	19
7-How old were you while starting use of computer?		
≤ 14	49.5	54
> 14	50.5	55
8-How did you familiarize with computer?		
Through experience	100	109
Through a special course	85.3	93
Through a course in the university	46.8	51
Others	3.7	4
9- Which computer activities do you use more?		
Internet	60.6	66
Word	33	36
Multimedia	27.5	30
Medline	14.7	16
Others	11	12
10- How frequently do you use the computer?		
Every day	33.3	36
2-3 days a week	46.7	50
Once a week	12.4	14
Once a month	7.6	9

**Table-II:** Frequency of medical students regarding internet access

**Questions**	**%**	**No**
1-Do you have access to internet?		
Yes	100	109
2-If yes, please answer the following		
At university	94.5	103
At home	82.6	90
Internet cafe	15.6	17
Others	9.2	10
3- How would you describe the access of the internet?		
Very good	31.2	34
Good	34.9	38
Moderate	21.1	23
Poor	9.2	10
Very poor	0.9	1
No response	2.7	3
4- How quick do you think it is to use?		
Very quick	4.7	5
Fairly quick	33.6	36
Average	45.8	49
Not very quick	9.3	10
Not at all quick	6.5	7
No response	1.8	2
5- How confident are you with regard to the accuracy of information on the internet?		
Very confident	19.3	21
Fairly confident	69.7	76
Not at all confident	9.2	10
No response	1.8	2
6-Do you have e-mail address?		
Yes	90.8	99
No	9.2	10
7- Do you have access to wireless at home?		
Yes	20.2	22
No	79.8	87
8- What features of internet do you most frequently use?		
Medical literature search	95.4	104
Mail	74.3	81
Leisure	62.4	68
General information	56.9	62
Chat	19.2	21
Others	22.9	25
All	11	12

**Table-III:** Percentage of students regarding how frequently they access medical subject in English-language and Farsi-language sites

***Internet use***	***English Sites (%)***	***Farsi Sites (%)***
Very Frequent	17.4	9.2
Frequent	50.5	53.2
Rare	22	29.4
Very rare	8.3	5.5
Not at all	1.8	2.7

In our university, computer courses are not taught and students learn computer privately. Based on our study, 46.8% of students learned computer in out of university course, but only 6.3% of Jordanian students learnt it out of academic institutions and 21% of them learnt it through academic courses and remaining learnt computer experimentally. So these results had significant difference compared to our study.^10^

In response to this question: what types of computers facilities do you use: 60.5% answered internet and 33% word processing but these percentages are 82.5% for internet and 22.4% for word processing in Jordanian study? ^10^ In our study, most of students (46.7%) were using computer 2-3 times weekly but in Jordanian students 48.1% of them were using computer once a month. Comparing these two studies indicated: more computer use among Shahid Sadoughi University of Medical Sciences medical students.

In our study, 94.5% of students used internet in university and 82.6% used internet in their homes. Compared to ours the study performed in Jordan showed 100% of student used internet in university and 54% used internet in their homes, which indicates that Iranian students had more access to internet at home. (Also in English study 53% of students used internet at home).^10^^,^^11^

In our study only 32.1% of students believed that they had very good access to internet while this belief was different among boys and girls (PV=0.025), 73.5% of girls and 26.5% of boys believed that internet accessibility was very good which reveals that boys are more demanding for high speed internet.

In this study, only 19.3% of students were very confident abut accuracy of internet contents, whereas in England and Jordan these percents were 29% and 12.7% respectively, which indicates various confidence levels to accuracy of internet contents.^10^^,^^11^ In our study, 90.8% of students had E-mail address which was similar to Moroccan students (92.9%).^6^

In Morocco (20.8%) and in our studies (20.2%), the students had similar access to wireless and difference was not significant. In our study most of the students used internet for access to medical information's (95.4%). These results were 58% and 95.7% for English dental students and Moroccan medical students respectively.^6^^,^^11^ So our results were closer to Moroccan study. In our study, there was significant difference between girls (59.6%) and boys (40.4%) in internet use for general information's (PV= 0.023). In our study most of students (74.3%) used Google and Jordanian students (23%) used Yahoo motor search for searching.^10^

About using internet for scientific contents there was no significant difference between dental and medical students of Shahid Sadoughi University of Medical Science, Yazd, Iran. So both groups mostly used internet for scientific contents.^5^

When asked what problems they face about internet, slow speed of internet was mentioned by most of our students (43.7%) but in the English study, dental students’ response was lack of time, whereas Moroccan medical students stated less familiarity to internet use (63.8%).^5^^,^^6^^,^^11^

In this study 62.4% of students mostly used internet contents in Persian language and 67.9% mostly used internet contents in English language. Girls were using internet in Persian significantly more as compared to boys. (PV=0.042).

Use of dental contents in English form (67%) was more significant compared to dental contents used in Persian form (21%). Probably this difference could be due to more accessibility of Persian literature in medicine as against Persian literature in dentistry or may be better knowledge of English language in dental students.^5^

## CONCLUSION

Overall, the use of computer and internet by medical students was good but because of English contents on the internet (medical or non medical) we suggest to improve the English language skill in medical students of Shahid Sadoughi University of Medical Sciences. We also recommend to improve the internet speed (the most problem that medical students encountered during internet use) to overcome lack of time reported by medical students.

## Notes

***Source of funding: ***This work was supported by Shahid Sadoughi University of Medical Science, Yazd, Iran.
